# Non-targeted Metabolomics Profiling of Plasma Samples From Patients With Major Depressive Disorder

**DOI:** 10.3389/fpsyt.2021.810302

**Published:** 2022-02-21

**Authors:** Zhonghao Wu, Heming Yu, Yu Tian, Yue Wang, Yong He, Tianlan Lan, Yan Li, Mengge Bai, Xiangyu Chen, Zhi Chen, Ping Ji, Hongmei Zhang, Xin Jin, Jinlin Song, Ke Cheng, Peng Xie

**Affiliations:** ^1^NHC Key Laboratory of Diagnosis and Treatment on Brain Functional Diseases, The First Affiliated Hospital, Chongqing Medical University, Chongqing, China; ^2^State Key Laboratory of Ultrasound in Medicine and Engineering, College of Biomedical Engineering, Chongqing Medical University, Chongqing, China; ^3^Department of Neurology, The First Affiliated Hospital, Chongqing Medical University, Chongqing, China; ^4^Institute of Neuroscience, Basic Medical College, Chongqing Medical University, Chongqing, China; ^5^The M.O.E. Key Laboratory of Medical Diagnostics, The College of Laboratory Medicine, Chongqing Medical University, Chongqing, China; ^6^Chongqing Key Laboratory for Oral Diseases and Biomedical Sciences, The Affiliated Hospital, Stomatology of Chongqing Medical University, Chongqing, China; ^7^College of Stomatology, Chongqing Medical University, Chongqing, China; ^8^Key Laboratory of Psychoseomadsy, Stomatological Hospital, Chongqing Medical University, Chongqing, China; ^9^Chongqing Municipal Key Laboratory of Oral Biomedical Engineering of Higher Education, Chongqing, China

**Keywords:** non-targeted metabolomics, major depressive disorder, biomarkers, plasma, LC-MS/MS, receiver operating characteristic curve, diagnosis

## Abstract

**Background:**

Major depressive disorder (MDD) is a neuropsychiatric disorder caused by multiple factors. Although there are clear guidelines for the diagnosis of MDD, the direct and objective diagnostic methods remain inadequate thus far.

**Methods:**

This study aims to discover peripheral biomarkers in patients with MDD and promote the diagnosis of MDD. Plasma samples of healthy controls (HCs, *n* = 52) and patients with MDD (*n* = 38) were collected, and then, metabolism analysis was performed using ultrahigh-performance liquid chromatography–tandem mass spectrometry (LC–MS/MS). Heatmap analysis was performed to identify the different metabolites. Meanwhile, receiver operating characteristic (ROC) curves of these differential metabolites were generated.

**Results:**

Six differential metabolites were found by LC–MS/MS analysis. Three of these were increased, including L-aspartic acid (Asp), diethanolamine, and alanine. Three were decreased, including O-acetyl-L-carnitine (LAC), cystine, and fumarate. In addition, LAC, Asp, fumarate, and alanine showed large areas under the curve (AUCs) by ROC analysis.

**Conclusion:**

The study explored differences in peripheral blood between depressed patients and HCs. These results indicated that differential metabolites with large AUCs may have the potential to be promising biomarkers for the diagnosis of MDD.

## Introduction

Major depressive disorder (MDD) is a severe neuropsychiatric disorder, causing a dark mood, inferiority complex, disability, and even suicide, and seriously affects the quality of life of the patients (1). According to the prediction of the World Health Organization (WHO), MDD will become a major cause of disability worldwide by 2030 (2). Through in-depth studies of MDD, diverse hypotheses regarding the cause have been put forward, such as neurotransmitter (NT) imbalance (3), neurogenesis abnormality (4), hypothalamic–pituitary–adrenal axis dysfunction (5), and gut microbiota disturbance (6). Although many studies regarding the etiology and pathogenesis of MDD have made great progress, effective touchstones remain urgently needed for disease diagnosis.

During the past several years, numerous biomarkers in peripheral body fluids and the central nervous system were found in our previous studies. Within peripheral blood mononuclear cells, some metabolites are considered biomarkers, such as γ-aminobutyric acid and dopamine, with functions that serve as NTs (7). Moreover, a variety of crucial metabolites were found in urine, including a series of important amino acids. Meanwhile, a correlation between sex and metabolites was illustrated in a previous study (8). As the understanding of relevant metabolites has increased, a growing number of biomarkers have improved the accuracy and specificity of the diagnosis of MDD.

Plasma biomarkers provide a line of direct and effective diagnostic evidence for MDD. On the basis of several studies evaluating the peripheral blood of depressed patients, a meta-analysis has revealed that various NT-related metabolites contribute to the diagnosis of MDD (9). However, the existing targeted metabolomic approaches related to NTs fail to detect sufficient potential metabolites. Thus, a new non-targeted metabolomic approach, applied to improve detection sensitivity, is needed.

To identify eligible molecules that promote measurement reliability, we conducted non-targeted liquid chromatography–tandem mass spectrometry (LC–MS/MS) to further identify biomarkers in patients with MDD. In addition, we analyzed the relationships among differential metabolites and correlations between metabolites and sex. Our results, as a supplement to previous research findings, provide new evidence for the diagnosis of MDD and possible treatment targets.

## Materials and Methods

### Subjects

All subjects were voluntarily recruited from the First Affiliated Hospital of Chongqing Medical University. The diagnostic criteria of the DSM-IV and the Hamilton depression rating scale (HDRS) were assessed for a single depressed episode and the severity of patients with MDD, respectively. Only depressed patients who gained HDRS scores greater than or equal to 17 were enrolled. Depressed subjects with confounding factors such as other mental disorders, history of mental illness, illegal drug use, and major physical defects were excluded from this study. All depressed subjects were first-onset, drug-naïve, and depressed patients. The healthy controls (HCs) were normal clusters from the physical examination center without the interference factors mentioned above. All experimental protocols were approved by the Ethics Committee of Chongqing Medical University. In addition, a prime principle of all experiments was in line with the ethics of the World Medical Association (Declaration of Helsinki). All methods were carried out in accordance with the relevant regulations. Informed consent was obtained from all subjects.

### Human Plasma Collection

Blood samples were collected into 10-ml anticoagulant tubes with ethylenediaminetetraacetic acid, and, immediately, all sample tubes were homogenized upside down. Afterward, all blood specimens were centrifuged (1,000 g for 15 min at room temperature). Then, the resultant plasma samples were separated and stored at −80 °C before pretreatment with LC–MS/MS.

### LC–MS/MS Analysis

LC–MS/MS analysis was performed as described previously (10). In brief, each sample was diluted in 400 μl of a mixture of methanol and acetonitrile (a volume ratio of 1/1) with dichlorotoluene (0.3 mg/ml, 5%) as the internal standard. After vortexing for 1 min, samples were ultrasonicated on ice for 5 min and allowed to stand for 30 min. Then, 300 μl of supernatant was collected after centrifugation (10,000 g, 4°C for 10 min). The extraction was successively diluted with equal amounts of chloroform and 100 μl of distilled water followed by vortexing for 30 s, ultrasonication on ice for 5 min, and standing for 30 min. Ultimately, followed by centrifugation (10,000 g, 4 °C for 10 min), 150 μl of supernatant of each sample was collected. A Nexera ultrahigh-performance LC (UPLC) system (Shimadzu, Kyoto, Japan) and a tandem Q Exactive plus MS system (Thermo Scientific™) were used to dissociate and analyze the metabolites.

### Metabolite Data Analysis

Compound Discoverer 3.0 and Simca-p 14.1 software programs were used to process metabolite data. Supplementary Figure 1 shows the basic peak ion flow diagram of the quality control samples in positive and negative ion modes. These quality control results indicated that the methodology and sample quality were qualified. The Human Metabolome Database (HMDB) was applied to search for differential metabolites. Besides HMDB, we have also used self-built standers to assist in the characterization of these metabolites. Unsupervised multivariate statistical analysis (PCA) and orthogonal partial least-squares discriminant analysis (OPLS-DA) were used to reflect overall metabolic differences and variation between groups. To verify the accuracy of the OPLS-DA model, a 200-iteration permutation test was conducted to eliminate the contingency of supervised learning methods. We screened metabolites that scored >60 to obtain reliable outcomes. Only variable importance plots (VIPs) >1 and significance *p* < 0.05 were considered differential metabolites.

### Statistical Analysis

All the results in this research are presented as the Means ± S.E.M., and all data were analyzed in SPSS 21.0 software. The Chi-square test was used for sex statistics. A two-tailed Student's *t*-test was applied to data obeying normal distribution, and the Mann–Whitney *U*-test was used to data characterized by abnormal distribution. Adjusted *p*-values were calculated for metabolites by the Bonferroni method (HOLM). Pearson correlation was used to analyze correlations among these differential metabolites. Spearman's correlation was applied to analyze the correlations between the clinical index and metabolites in the MDD cohort. Receiver operating characteristic (ROC) curve analysis was performed to determine the diagnostic value of differential metabolites. Significance was defined as ^*^p < 0.05, ^**^p < 0.01, and ^***^p < 0.001.

## Results

### Clinical Information of Recruited Subjects

On the basis of the above criteria, 52 HCs and 38 patients with MDD were recruited into this study (Table 1). The HDRS scores of all depressed patients were greater than or equal to 17. Other factors, including sex and age, were not significantly different between the HC and MDD groups.

**Table 1:** Demographic details of recruiters.

	**HC**	**MDD**	**P**
Sample size	52	38	—
Sex (M/F)	27/25	13/25	0.10
Age (year)	24.08 ± 0.21	25.71 ± 0.85	0.18
HDRS	—	21.74 ± 0.70	—

*HC, healthy controls; MDD, major depressive disorder; M/F, male/female; HDRS, Hamilton depression rating scale. Data are represented as the mean ± s.e.m. Chi-squared test for classified variables (sex); two-tailed student's t-test for age*.

### Metabolomics Analysis of Plasma Samples From HC and MDD

**Figure 1:**
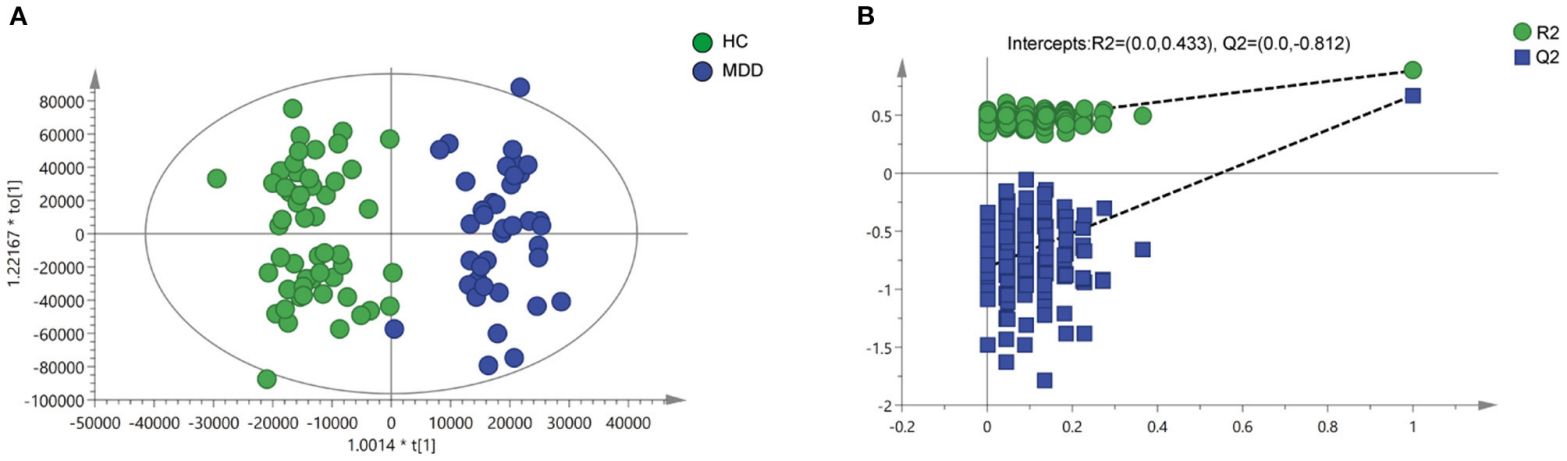
Metabolomics analysis of plasma samples from HCs and patients with MDD. **(A)** Discriminant analysis of the orthogonal partial least squares (OPLS-DA) method (R^2^X = 0.796, R^2^Y = 0.883, Q^2^ = 0.671). **(B)** Permutation tests performed with 200 random permutations in OPLS-DA models showing R^2^ (green circles) and Q^2^ (blue squares) values from the permuted analysis (left) as significantly lower than the corresponding original R^2^ and Q^2^ values (right) (R^2^ = 0.433, Q^2^ = −0.812).

OPLS-DA analysis was used to explore the metabolic differences between 52 HCs and 38 first-onset, drug-naïve, depressed patients, and almost no overlap was found (R^2^X cum = 0.796, R^2^Y cum = 0.883, Q^2^ cum = 0.671; Figure 1A). It could be seen from the parameters of R^2^X, R^2^Y, and Q^2^ that the OPLS-DA model had strong explanatory and predictive power. Meanwhile, permutation tests were performed to validate the OPLS-DA model. The intercept of R^2^ was 0.433 and that of Q^2^ was −0.812 (Figure 1B).

### Differential Metabolites in Plasma of HC and MDD

**Figure 2:**
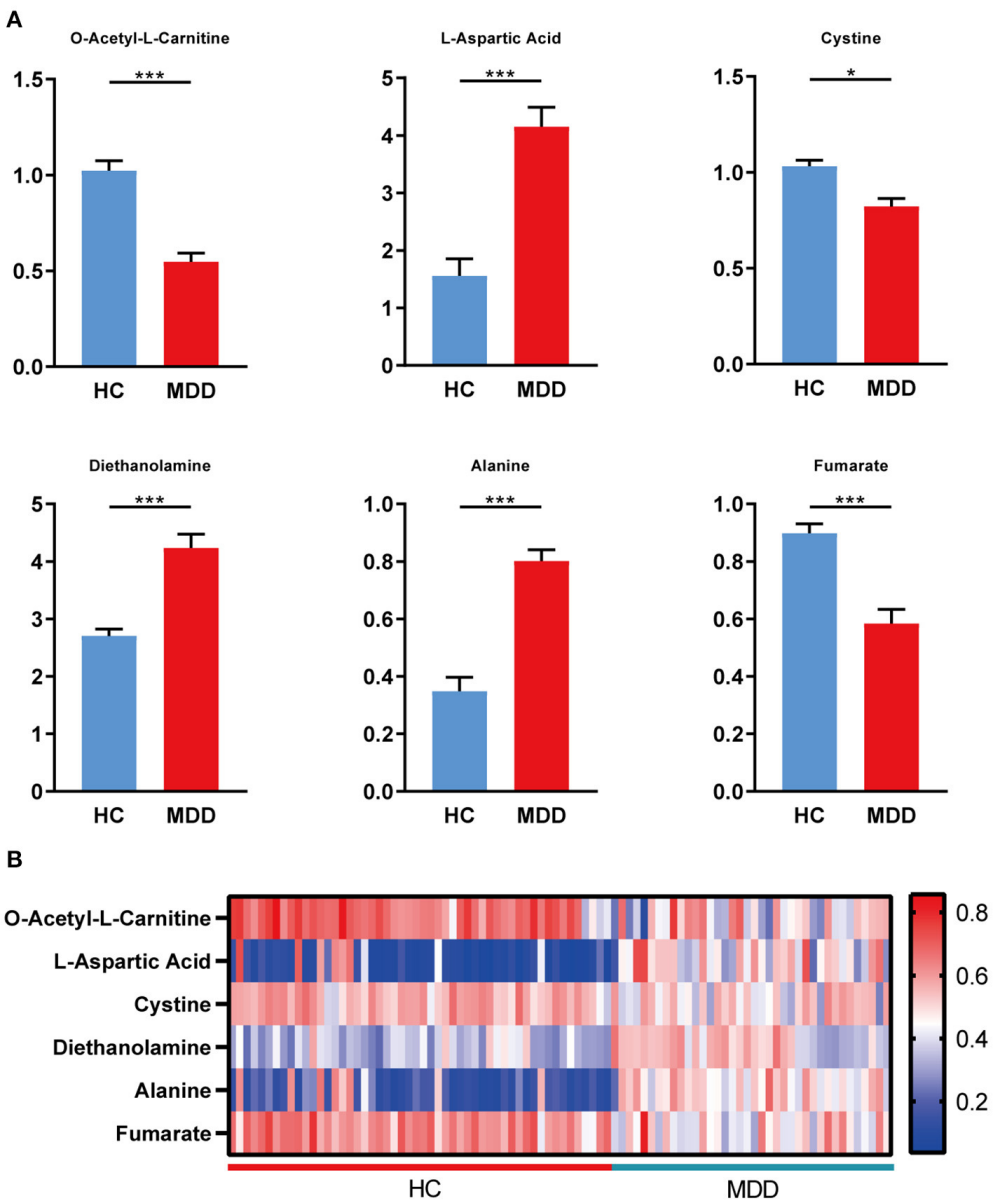
Differential metabolites in plasma of HC and MDD. **(A)** Levels of metabolites were determined by LC–MS/MS, including LAC, Asp, cystine, diethanolamine, alanine, and fumarate, in the HC group (*n* = 52) and the MDD group (*n* = 38). **(B)** Heatmap of the differential metabolites in plasma (HC vs. MDD). Blue, positive correlation; red, negative correlation. Student's *t*-test and Mann–Whitney *U*-test were used for the comparison of two groups in which data obeyed normal and abnormal distribution, respectively. ^*^p < 0.05, ^**^p < 0.01, and ^***^p < 0.001.

Differential metabolites in the HC group vs. the MDD group were filtered by VIP > 1 and *p* < 0.05. Ultimately, a total of six identified metabolites were regarded as significantly different in plasma (Table 2). In patients with MDD, three of these differential metabolites were increased (Asp, diethanolamine, and alanine), and three were decreased (LAC, cystine, and fumarate). The relative concentrations of the six identified metabolites are shown in Figure 2A. Then, a heatmap was created to visually show the relative changes in these metabolites (Figure 2B).

**Table 2:** The plasma metabolites responsible for discriminating MDD subjects from HCs.

**Metabolites**	**RT [min]^†^**	**Score^**‡**^**	**VIP value^**§**^**	**Holm P^**¶**^**	**FC^***††***^**
O-Acetyl-L-Carnitine	5.96	99.5	14.88	1.116E-06	0.53
L-Aspartic Acid	8.38	98.6	3.44	5.255E-05	2.66
Cystine	10.95	89.1	2.72	0.026689	0.80
Diethanolamine	5.92	98.5	2.69	6.617E-06	1.57
Alanine	6.32	66.5	1.49	2.578E-07	2.30
Fumarate	8.79	83.2	1.16	0.0001192	0.65

† *Retention time (RT): The time that the metabolite flows out of the chromatography after separation of a complex substance by chromatography*.

‡ *Score: When the software qualitatively matches the precise mass number, secondary fragments, retention time, isotope distribution, and other parameters, the comprehensive score value is obtained*.

§ *Variable importance in the projection (VIP) was obtained from OPLS-DA with a threshold of 1.0*.

¶ *Holm p was obtained by two-tailed Student's t-test followed by Bonferroni method (HOLM)*.

†† *Fold change (FC): Values greater than 1 indicate higher levels in depressed patients relative to healthy controls; values less than 1 indicate lower levels in depressed patients relative to healthy controls*.

### Correlation Analysis

**Figure 3:**
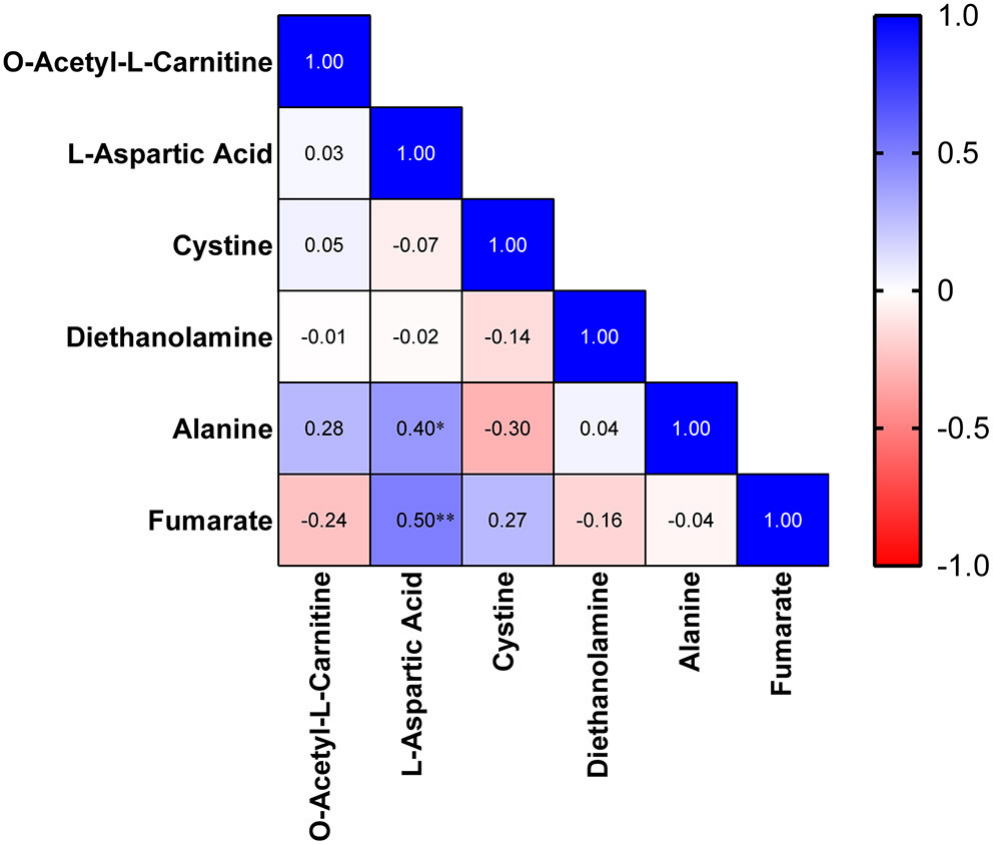
Heatmap of the correlation matrix among differential metabolites.

Then, we analyzed the correlations between the clinical index and metabolites. In the MDD cohort, Asp was found to be correlated with sex (r: −0.362, *p* = 0.026). Furthermore, a strong correlation was identified between sex and alanine (r: −0.443, *p* = 0.005). Meanwhile, we analyzed the correlations among these differential metabolites in the MDD cohort. A heatmap of the correlation matrix showed that Asp had positive correlations with alanine (r: 0.400, *p* = 0.013) and fumarate (r: 0.502, *p* = 0.001; Figure 3).

### Judgment of Diagnostic Value of Differential Metabolites

**Figure 4:**
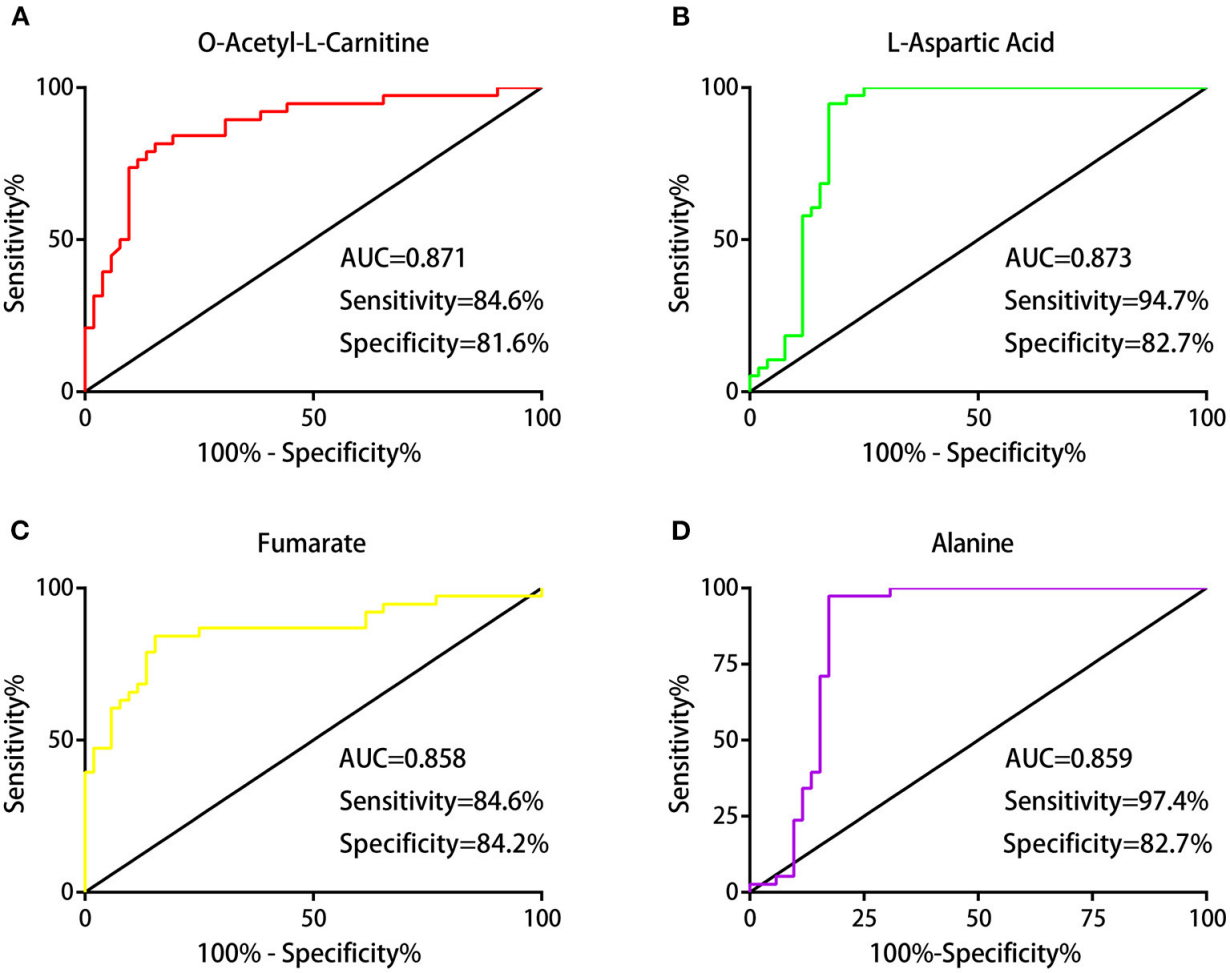
Judgment of the diagnostic value of differential metabolites. Receiver operating characteristic (ROC) analysis showing the diagnostic performances of these differential metabolites: the areas under the curve (AUCs) values of LAC, Asp, fumarate, and alanine were 0.871, 0.873, 0.858, and 0.859, respectively **(A–D)**.

To evaluate the diagnostic value of these differential metabolites, we generated ROC curves. Four of the differential metabolites had high diagnostic value, including LAC (AUC, 0.871; sensitivity, 84.6%; specificity, 81.6%), Asp (AUC, 0.873; sensitivity, 94.7%; specificity, 82.7%), fumarate (AUC, 0.858; sensitivity, 84.6%; specificity, 84.2%), and alanine (AUC, 0.859; sensitivity, 97.4%; specificity, 82.7%; Figures 4A–D).

## Discussion

In this research, we performed non-targeted metabolomics using LC–MS/MS to analyze the differential metabolites. Subsequently, we executed a series of analyses of these differential metabolites, including heatmaps, correlations, and diagnostic value assessments, and found several metabolites with high diagnostic value.

Our results revealed four metabolites with high diagnostic value, including LAC, Asp, alanine, and fumarate. LAC is a key substance of fatty acids in the oxidative metabolic pathway that allows fatty acids to enter mitochondria. In addition, there is evidence that LAC can freely cross the blood–brain barrier (11). Previous reports demonstrated that abnormal fatty acid metabolism was associated with depression (12). A clinical study showed that the plasma concentration of LAC in patients with MDD was lower than that in HCs; moreover, the decrease was even greater in patients with treatment-resistant depression. In addition, LAC acted as a rapid antidepressant after supplementation *via* epigenetic induction of type 2 metabolized glutamate (mGlu2) receptors and promotion of hippocampal neurogenesis (13–15). Asp is an important component in the synthesis of excitatory NTs. In this process, the amino group of Asp was transferred to glutamate in an aspartate aminotransferase reaction (16), and it was the main nitrogen donor for glial glutamate synthesis (17). It was reported that patients with MDD suffered dysfunction of NTs and synaptic transmission (18, 19). Asp could induce changes in H^+^ and Na^+^ in rat hippocampal astrocytes and regulate excitatory synaptic transmission in the hippocampal CA1 region of rats (20, 21). Alanine is a nonessential amino acid. Previous studies reported that alanine levels were increased in the serum of patients with MDD compared to HCs (22), which was consistent with our outcomes. In addition, mice treated with ketamine exhibited reduced alanine levels (23). The in-depth mechanism of the association of alanine with depression remains to be defined and requires further exploration. Fumarate could influence gene expression associated with neuroprotection, including in microglia (24) rather than astrocytes (25). Similarly, fumarate could upregulate nuclear factor-like 2–dependent antioxidant reactions and ultimately protect against inflammation and oxidative stress in neurons and astrocytes (26, 27). In previous studies, fumarate was scarcely associated with MDD. Therefore, it is of great significance to explore whether fumarate could be a new biomarker for the diagnosis of MDD.

We identified six differential metabolites in the plasma of patients with MDD by LC–MS/MS analysis. On the basis of a non-targeted metabolomic approach, our research provides more evidence for effective promising diagnostic biomarkers of MDD from a more extensive database of metabolites. These metabolites hope to complement and extend the clinical testing methods of MDD.

## Limitation

There are some limitations in this study. 1) These recruiters were from same region and nationality; thus, our data need further integrated analysis with more studies from other regions and ethnic groups. 2) Our study did not perform further validation, so other new recruiters need to be involved in future studies to enhance the reliability of our findings.

## Data Availability Statement

The datasets generated and/or analyzed during the current study are not publicly available because the datasets relate to other unpublished projects, but are available from the corresponding author on reasonable request.

## Ethics Statement

All experimental protocols were approved by the Ethical Committee of Chongqing Medical University. Besides, a prime principle of all experiments was in line with the ethics of the World Medical Association (Declaration of Helsinki). All methods were carried out in accordance with relevant regulations. Informed consent was obtained from all subjects.

## Author Contributions

ZW wrote the main manuscript text. ZW, HY, YT, YW, YH, TL, YL, MB, XC, and ZC were responsible for clinical specimen collection. ZW and HY were responsible for statistics and all figures. PJ, HZ, XJ, and JS modified the manuscript. KC conceptualized this subject. PX provided the funding and checked the manuscript. All authors reviewed the manuscript.

## Funding

Our research was supported by the National Key R&D Program of China (grant no. 2017YFA0505700), the Non-profit Central Research Institute Fund of Chinese Academy of Medical Sciences (grant no. 2019PT320002), the National Key Program International Cooperation Project (grant no. 81820108015), and the National Natural Science Foundation of China (grant no. 81701361).

## Conflict of Interest

The authors declare that the research was conducted in the absence of any commercial or financial relationships that could be construed as a potential conflict of interest.

## Publisher's Note

All claims expressed in this article are solely those of the authors and do not necessarily represent those of their affiliated organizations, or those of the publisher, the editors and the reviewers. Any product that may be evaluated in this article, or claim that may be made by its manufacturer, is not guaranteed or endorsed by the publisher.
