# Supplementary figures and images for: Non-targeted Metabolomics Profiling of Plasma Samples From Patients With Major Depressive Disorder
Source: Front Psychiatry. 2022 Feb 21;12:810302. doi: 10.3389/fpsyt.2021.810302 (PMC8899025; doi:10.3389/fpsyt.2021.810302)

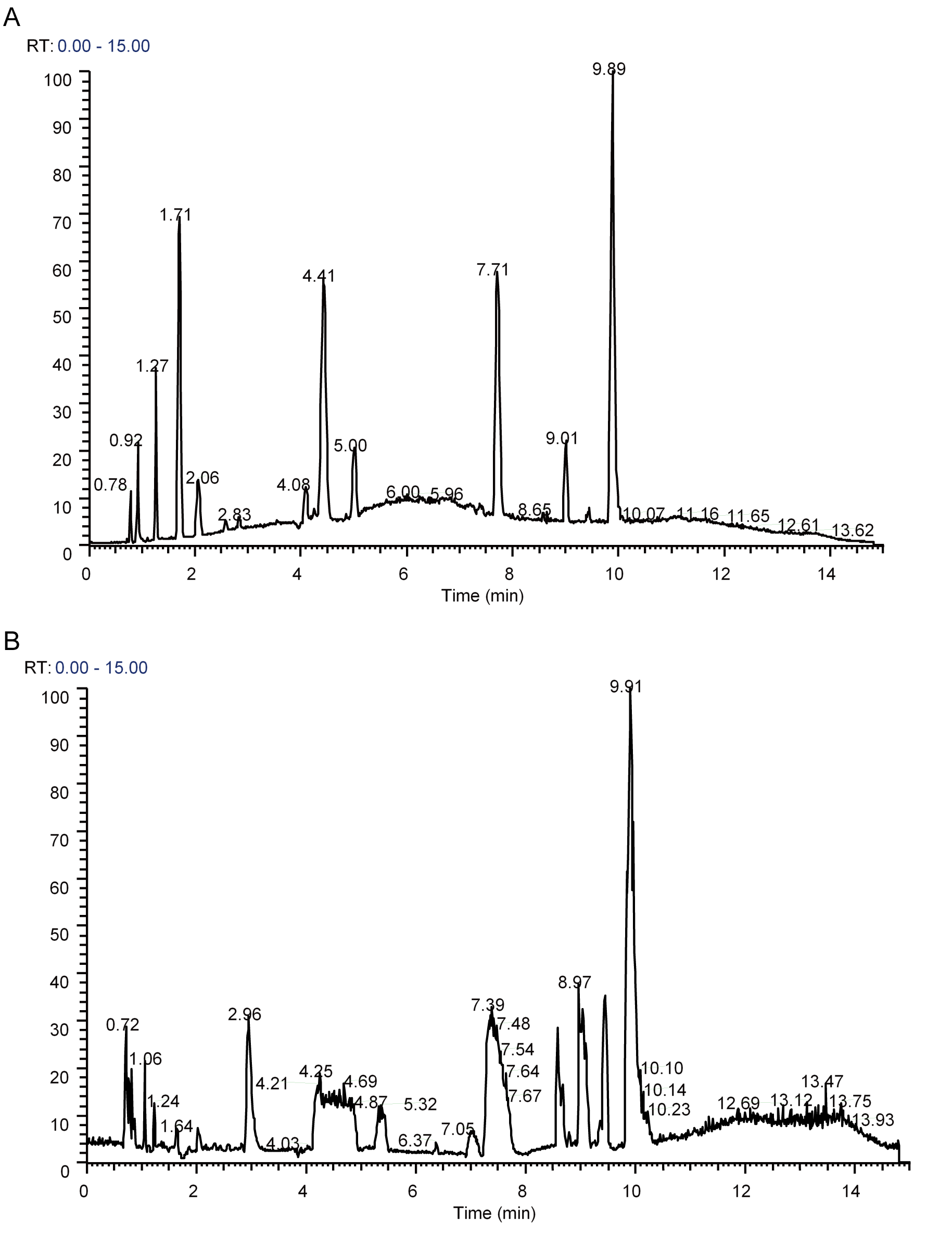

Supplement: Supplementary file 1 [file Image_1.TIF]
