# Supplementary material for: Non-targeted Metabolomics Profiling of Plasma Samples From Patients With Major Depressive Disorder
Source: Front Psychiatry. 2022 Feb 21;12:810302. doi: 10.3389/fpsyt.2021.810302 (PMC8899025; doi:10.3389/fpsyt.2021.810302)
Supplement: Supplementary file 2 [file Data_Sheet_1.docx]

**Supplementary figure 1. The basic peak ion flow diagram of the quality control samples.**

The basic peak ion flow diagram of the quality control samples in positive ion modes (A). The basic peak ion flow diagram of the quality control samples in negative ion modes (B).
